# Advances in Maize Transformation Technologies and Development of Transgenic Maize

**DOI:** 10.3389/fpls.2016.01949

**Published:** 2017-01-06

**Authors:** Pranjal Yadava, Alok Abhishek, Reeva Singh, Ishwar Singh, Tanushri Kaul, Arunava Pattanayak, Pawan K. Agrawal

**Affiliations:** ^1^Indian Council of Agricultural Research – Indian Institute of Maize ResearchNew Delhi, India; ^2^International Centre for Genetic Engineering and BiotechnologyNew Delhi, India; ^3^Indian Council of Agricultural Research – Vivekananda Parvatiya Krishi Anusandhan SansthanAlmora, India; ^4^Indian Council of Agricultural Research – National Agricultural Science FundNew Delhi, India

**Keywords:** maize, transformation, transgenics, tissue culture, gene editing

## Abstract

Maize is the principal grain crop of the world. It is also the crop where genetic engineering has been employed to a great extent to improve its various traits. The ability to transform maize is a crucial step for application of gene technology in maize improvement. There have been constant improvements in the maize transformation technologies over past several years. The choice of genotype and the explant material to initiate transformation and the different types of media to be used in various stages of tissue culture can have significant impact on the outcomes of the transformation efforts. Various methods of gene transfer, like the particle bombardment, protoplast transformation, *Agrobacterium*-mediated, *in planta* transformation, etc., have been tried and improved over years. Similarly, various selection systems for retrieval of the transformants have been attempted. The commercial success of maize transformation and transgenic development is unmatched by any other crop so far. Maize transformation with newer gene editing technologies is opening up a fresh dimension in transformation protocols and work-flows. This review captures the various past and recent facets in improvement in maize transformation technologies and attempts to present a comprehensive updated picture of the current state of the art in this area.

## Introduction

Maize (*Zea mays* L.) or corn is the principal crop of the world and stands first among the grain crops in terms of production. It is primarily used as animal feed and raw materials for various industries, and only a minor proportion is used as direct human food. The ever increasing human population and consumption of animal-derived foods is leading to enhanced demand for maize grains. However, various biotic and abiotic stresses are the bottleneck in enhancing maize production, productivity, and quality in the limited cultivable land available. To overcome these challenges, genetic engineering of maize with desired target genes have been extensively employed to produce transgenic maize cultivars with improved traits. The first transgenic maize cultivars were launched commercially 20 years ago in 1996 in the USA. Since then, maize has become the main target crop for plant genetic engineering. Among all crops, maize has the highest number of transgenic events that have been commercialized. Thus, transformation to develop transgenic maize has been a forefront technology for the genetic improvement of this crop.

## Choice of Genotypes and Explants for *In Vitro* Regeneration and Transformation

The selection of a particular maize genotype for transformation is the first and a crucial step in maize transformation. Usually, the selection of the genotype is not primarily dependent upon its agronomic superiority, but on its amenability for tissue culture and transformation. Once the desired gene is introduced in a tissue culture friendly genotype, it can be transferred much conveniently to any agronomically superior genotype through marker assisted conversion. Tomes and Smith (1985) and Hodges et al. (1986) suggested that regeneration capacity of maize genotypes in tissue culture was genetically determined by nuclear genes. Further, Willman et al. (1989) indicated that at least one gene or a block of genes influenced somatic embryogenesis of maize in tissue cultures. Bohorova et al. (1995) also described the effect of the different genotypes on somatic embryogenesis capacity.

Hi-II (High type II callus production) is one of the most widely used genotype for commercial maize transformation. Callus induction is a critical step in maize transformation. Most maize genotypes produce a less compact and less regenerable type of callus termed as Type I callus. On the other hand, Type II calluses are friable, embryogenic, and transformable (Green, 1982; Armstrong et al., 1991; Hansen and Wright, 1999). Interestingly, Hi-II is an F_1_ hybrid line. Its parents (“Hi-II Parent A” and “Hi-II parent B”) were derived from F_2_ population of A188 X B73 cross through four generations of tissue culture, selfing and sib-pollinations. The immature embryo explants from Hi-II have been reported to give 100% Type II response (Armstrong and Green, 1985). Hi-II genotype showed maximum transformation efficiency with a range of 12–18% (Vega et al., 2008), while with A188, occasionally, 30% transformation efficiency with *Agrobacterium*-mediated method has been reported (Ishida et al., 1996). An Egyptian genotype- Line Gz 643 exhibited 42.2% regeneration frequency (El-itriby et al., 2003). Among tropical maize genotypes, IL3 was found to be most amenable to transformation and proved to be superior to A188 temperate genotype with a transformation frequency of 31.7 and 5.82%, respectively (Rasha et al., 2013). Embryogenic callus initiated from immature embryos and cell suspension cultures of embryogenic callus are most preferred targets for maize transformation (Armstrong, 1999; Hansen and Wright, 1999; Torney et al., 2007). Apart from immature embryos from Hi-II, a number of other genotypes and various other explants have also been used with varying success (**Table 1**). It is interesting to note that few other genotypes, have been reported to exhibit transformation frequencies better than Hi II, yet the latter has remained popular for commercial transformation. This might be due the fact that, apart from a moderately higher transformation frequency, Hi II transformed plantlets would exhibit vigorous growth as it is essentially an F_1_ hybrid.

**Table 1:** Summary of genotype, explant, media, and transformation methods used in various maize transformation studies.

S.No.	Genotype	Explant	Media	Transformation method	Gene	Transformation frequency	Reference
1	HKI 163	Seedling plumule	N6	*Agrobacterium-*mediated *in planta* transformation	*cry1Ab;bar*; *gus*	4%	Abhishek et al. (2014b)
2	Pa91 X H99 hybrid	Leaf	N6 and KT	Biolistic transformation	phosphomannose isomerase (pmi) promoter CMPS	NA	Ahmadabadi et al. (2007)
3	Tropical lines CML72, CML216, CML323, CML327, CML72, CML216, CML67, CML216, CML216, CML72.	Immature embryos	N6 basal medium	Biolistic transformation	ubi:*cry1ab*+35s:*bar-gus* ubi:*cry1ac*+35s:*bar-gus*	1–2%	Bohorova et al. (1999)
4	H99, A188, Pa91 X H99, A188 X H99	Immature embryos	N6, MSC, and MS	Biolistic transformation	*uidA; pat*	2–4%	Brettschneider et al. (1997)
5	AT-3	Pistil filaments		*Agrobacterium*-mediated	*gus; nptII*	NA	Chumakov et al. (2006)
6	Sweet corn	Aleurones and isolated embryos	MS	Biolistic transformation (Low-pressure BioWare gene gun)	*gus*	NA	Kao et al. (2008)
7	Inbred line H99 and Pa91	Immature embryo and Type I callus	N6	Electroporation	*nptII*	NA	D’Halluin et al. (1992)
8	Egyptian inbred lines Gz 643	Scutellar tissues of immature embryos (1.0–2.0 mm long)	N6-based media	Biolistic transformation	*gus; bar*	NA	El-itriby et al. (2003)
9	A188 X B73	Immature zygotic embryo	N6 and MS	Silicon carbide whisker-mediated transformation	*bar*	NA	Frame et al. (1994)
10	Hi-II	Type II callus	N6	Biolistic transformation	*bar*	0–11%	Frame et al. (2000)
11	HE/89 Embryogenic suspension culture (Ke2/2)	Embryogenic protoplasts	N6	PEG mediated direct DNA transfer	mutant dihydrofolate reductase *(dhfr)*	NA	Golovkin et al. (1993)
12	Hi-II and inbreds P38 and N46	Immature embryo	560P medium and 560Y	Microprojectile bombardment and *Agrobacterium-*mediated transformation	*RepA; gfp; bar*	3–20%	Gordon-Kamm et al. (2002)
13	A188 X B73 A188 X B84	Type II callus of immature embryo	N6	Microprojectile bombardment	*gus; bar*	NA	Gordon-Kamm et al. (1990)
14	LH198 X Hi-II	Embryos	NA	*Agrobacterium-*mediated transformation	*cp4 epsps*	NA	Huang et al. (2004)
15	A188, W117, W59E, A554, W153R’H99, BMS cultivar	Immature embryo	N6	*Agrobacterium tumefaciens-*mediated	*gus; bar*	5–30%	Ishida et al. (1996)
16	Tropical inbred line Cat-100-6	Immature embryos	ML1	Biolistic transformation	*gus*	NA	Kemper et al. (1996)
17	Black Mexican Sweet (BMS; ATCC No 54022)	Intact maize cells in suspension culture	MS	Biolistic transformation	*npt II; gus*	NA	Klein et al. (1989)
18	Hi-II	Immature embryos	N6	*Agrobacterium*	*gus; bar*	NA	Miller et al. (2002)
19	W506, M37W	Immature embryos	MS and N6	Particle bombardment	*gus; bar*	NA	O’Kennedy et al. (2001)
20	Hi-II	Immature embryos	MS	Silicon carbide whisker mediated	*bar; uidA*	NA	Petolino et al. (2000)
21	Tropical inbred lines L3 and L1345	Immature embryos	N6	Biolistic transformation	*bar; uidA*	0.9–2.31%	Petrillo et al. (2008)
22	Hi-II	Coleoptiles	N6	Biolistic transformation	*gus*	2	Reggiardo et al. (1991)
23	Inbred H99	Immature embryos	N6	Biolistic transformation	*gus; nptII*	1–5%	Shiva Prakash et al. (2009)
24	Inbred H99	Immature embryos	N6	Biolistic transformation	*nptII*	42–54% 30–38%	Shiva Prakash et al. (2008)
25	Egyptian inbred lines Giza 650, Sids 34, Sids 62	Immature embryos	N6	Particle bombardment and *Agrobacterium*-mediated transformation	*hva1*; *bar*	1–2%	Assem et al. (2008)
26	A188 X B73	Type II calli	MS	Biolistic transformation	*gus; bar*	NA	Vain et al. (1993)
27	Hi-II A X Hi-II B F_2_	Immature embryos (F_2_)	N6	*Agrobacterium-*mediated	*gus; bar*	17.5%	Vega et al. (2008)
28	H99, LH198 X Hi II, PHA (Pa91 X H99)XA188, KHI and proprietary lines LI, L2, L4, L9, and L9 × L5	Seedling derived Type I callus	MS	*Agrobacterium-*mediated	CP4 modified *epsps; gfp; nptII*	2–11%	Vladimir et al. (2006)
29	A188 × B73	Immature embryo	N6	Biolistic transformation	*hpt*	NA	Walters et al. (1992)
30	Anther culture derived double haploid plants XH99 and FR16	Type I callus	N6 and Duncan’s medium	Biolistic transformation	*bar*; *uid*	NA	Wan et al. (1995)
31	Inbred lines X333, X301, X90 and Hybrid lines A1:Q31xZ3, B1:Q31XZ31, 6X41; 616X680	Immature embryo	N6	Biolistic transformation	s*b401*; *hpt*	0–7.3%	Wang et al. (2006)
32	CG00526	Type I calli derived from immature embryo medium	D callus induction	Biolistic transformation	*pmi*	45%	Wright et al. (2001)
33	Shen 137	NA	NA	Pollen-tube pathway *in planta* transformation	*gfp*	4.96%	Yang et al. (2009)
34	Hi-II	Embryogenic calli	NA	Biolistic transformation	*uidA; bar*	NA	Zhang et al. (1996)
35	Hi-II	Immature embryo	NA	*Agrobacterium-*mediated	*gus*	32.8–50.5%	Zhao et al. (1998)
36	Sweet corn genotypes HNP, IGES	Shoot tips	CSPD	Biolistic transformation	*bar; potato proteinase inhibitor II*	NA	Zhong et al. (1996)

*NA, not available.*

Recently, transgenic maize genotypes, especially suited for transformation have been developed. Transgenic maize overexpressing the maize Baby boom (*ZmBbm*) and maize Wuschel2 (*ZmWus2*) genes, showed high transformation frequencies in numerous previously non-transformable maize inbred lines (Lowe et al., 2016). Earlier, the maize streak virus replication-associated protein A (RepA) was shown to enhance transformation frequency and callus growth rate in maize transformation cycle. The RepA-containing transgenic maize calli were reported to remain embryogenic, readily regenerable, and capable of producing fertile plants that transmitted transgene expression in a Mendelian fashion (Gordon-Kamm et al., 2002). Similarly, overexpression of an embryo development related Lec1 gene in maize was shown to aid further re-transformation (Lowe et al., 2002).

*In vitro* regeneration in maize has been reported from immature embryos (Duncan et al., 1985; Bohorova et al., 1995; Ishida et al., 1996; Aguado-Santacruz et al., 2007), mature embryos (Huang and Wei, 2004; Al-Abed et al., 2006; Tiwari et al., 2015), nodal explants (Vladimir et al., 2006; Tiwari et al., 2015), leaf (Conger et al., 1987; Ahmadabadi et al., 2007), anthers (Ting et al., 1981; Barloy and Beckert, 1993), tassel and ear meristems (Pareddy and Petolino, 1990), protoplast (Mórocz et al., 1990), and shoot meristems (Sairam et al., 2003). Germinated split-seeds were also used as target tissue for callus formation and regeneration by Al-Abed in 2006. Abhishek et al. (2014a) analyzed the effect of using of immature embryos harvested from different Indian maize genotypes of different ages on callus induction, embryogenic Type II calli initiation and regeneration. The mean callus induction, embryogenic Type II calli production, and regeneration were found to be highest across all the genotypes in 12 days old immature embryos. The highest regeneration capacity was observed for the Indian maize genotypes- HKI 1105 and CM 300.

One of the major drawbacks of using immature embryos as explants is the limited availability of appropriate stage explant material. Recently, callus induction and regeneration of complete plants was demonstrated using split nodes derived from germinating mature embryos derived from sub-tropical maize (Tiwari et al., 2015). The VQL 2 genotype performed best with a regeneration frequency of 34% in callusing medium supplemented with 2.2 mg l^-1^ picloram and 0.5 mg l^-1^ 2,4-dichlorophenoxyacetic acid (2, 4-D). Significantly, transgenic maize overexpressing Wus2 and Bbm genes enabled direct *Agrobacterium*-mediated transformation of mature seed-derived embryo axes or leaf segments, without an intervening callus or meristem culture step (Lowe et al., 2016). The availability of a robust transformation protocol in tropical/sub-tropical maize genotypes using readily available explants like mature seed would be a major achievement toward engineering maize suitable for tropical and sub-tropical countries.

## Media for *In Vitro* Regeneration and Transformation

Optimization of culture medium components is most important for establishment of tissue culture. Green and Phillips (1975) produced the first somatic embryos in maize. They regenerated plants from embryo scutellar tissues which were initiated and maintained on Murashige Skoog (MS) media inorganic components, Straus media vitamins and amino acids, 20 g sucrose and 8 g agar per liter, and 2 mg l^-1^ 2,4-D. Establishment of robust *in vitro* regeneration system is a pre-requisite for venturing into maize transformation. Mostly, MS, N6 (Chu) or Linsmaier and Skoog (LS)-based culture media have been used for maize transformation at various stages of tissue culture. Optimization of different components, such as carbon source, amino acids, vitamins, and concentration of plant growth regulators in culture medium is often required while using these media. Different carbon sources (both reducing and non-reducing) have been used in the culture media depending upon genotypes and specific stages of growth. However, sucrose is most widely used carbon source. Even though cultured plant cells can synthesize amino acids themselves, a variety of amino acids, viz. *L*-glutamine, *L*-proline, *L*-asparagine, *L*-arginine, *L*-cysteine have been tested. The effects of various vitamins, *viz.* thiamine, riboflavin, niacin, pyridoxine, folic acid, pantothenic acid, biotin, ascorbic acid, myoinositol, etc., have also been tested. When using MS and N6 salts, lower nitrate and high NH_4_^+^ levels induce compact Type I callus, whereas, high nitrate level and low NH_4_^+^ level induce friable Type II callus (Elkonin and Pakhomova, 2000). The transformation efficiency in maize inbred lines can also be improved by optimizing MS and N6 salts (Frame et al., 2006). In culture medium, plant growth regulators play a critical role. Addition of AgNO_3_ to co-cultivation and callus induction media having 2,4-D, proline and casamino acids have shown induction of type II callus from immature embryos (Armstrong et al., 1991; Songstad et al., 1991; and El-itriby et al., 2003) (**Table 1**). In tropical maize genotypes, media, source of auxin, and their concentrations significantly influenced induction of callus (Rakshit et al., 2010).

## Transformation Techniques

### DNA Transfer to Protoplast by Electroporation

In this method, DNA of interest is transferred to protoplasts by applying electric pulse to the mixture of DNA and protoplasts. The first successful integration of transgene was performed in maize by transformation of Black Mexican Sweet maize protoplast by uptake of naked DNA through electrochemical method (Fromm et al., 1986). At that time, effective regeneration techniques in maize were not available, therefore full grown transformed plants could not be produced. The first full grown transgenic maize plants were developed in 1988 (Rhodes et al., 1988b). In 1980s, researchers tested different plant materials as explants for development of transgenic maize but only few instances became successful in regenerating whole plants from protoplasts (Rhodes et al., 1988a; Shillito et al., 1989). In order to obtain protoplasts for transformation by direct gene delivery, immature embryos became an excellent choice of explants (Rhodes et al., 1988a). In 1988, Rhodes et al. isolated protoplasts from embryogenic cell suspension culture of inbred line A188 and then transformed the protoplasts successfully by electroporation. The DNA of interest can also be transferred to the protoplasts by the addition of polyethylene glycol (PEG) to the mixture of protoplast and DNA instead of using electric pulse. Fertile transgenic maize plants of He/89 germplasm were obtained by using this method (Golovkin et al., 1993).

### Particle Bombardment

In this technique, target DNA is transferred through cell wall penetration by tungsten or gold particles coated with plasmid DNA. Highly accelerated coated particles are used to target into the desired tissue in maize which can be cell suspension culture, Type II callus, Type I callus, organogenic callus from seedlings, immature embryos or shoot meristem cultures. Among the monocotyledonous grains, maize has been one of the leading targets for genetic engineering through particle bombardment technique. In comparison to protoplast transformation, particle bombardment method generated more fertile transgenic events from embryogenic callus. Immature embryos were used as target tissue for particle bombardment mediated transformation of *cry1Ab* gene (Koziel et al., 1993). Ever since the initial development of biolistic transformation method, several improvements have been made. Songstad et al. (1996) developed a robust biolistic transformation protocol using Hi-II genotype. It was also observed that survival and transformation efficiency can be increased further, if immature embryos were pre-cultured prior to particle bombardment (Vain et al., 1993). Transformation frequency was found to be highly increased when immature embryos were cultured on high osmotic medium after particle bombardment (Brettschneider et al., 1997; El-itriby et al., 2003). A number of other studies have reported successful use of microprojectile bombardment technique for maize transformation (Klein et al., 1989; Fromm et al., 1990; Gordon-Kamm et al., 1990; Genovesi et al., 1992; Walters et al., 1992; Frame et al., 1994; Register et al., 1994; Wan et al., 1995; Brettschneider et al., 1997; Pareddy et al., 1997). An efficient biolistic transformation protocol for organogenic calli was also developed (O’Connor-Sánchez et al., 2002). Zhang et al. (2002) performed transformation of recalcitrant inbred lines B73 and PHTE4 by particle bombardment of shoot meristem culture. In maize, maximum number of commercial events deregulated, were produced using particle bombardment (**Table 2**).

**Table 2:** Methods of transformation employed in development of commercialized events of transgenic maize.

S. No.	Methods of transformation	Name of deregulated commercial event
1.	Chemically mediated introduction into protoplasts and regeneration	T14, T25
2.	Electroporation	MS3, MS6
3.	Microparticle bombardment of plant cells or tissue	676, 678, 680, Bt11 (X4334CBR, X4734CBR), Bt176 (176), CBH-351, DBT418, DLL25 (B16), GA21, LY038, MON801 (MON80100), MON802, MON809, MON810, MON832, MON863, NK603, TC1507
4.	Whiskers-mediated plant transformation	DAS40278
5.	*Agrobacterium tumefaciens*-mediated plant transformation	32138, 3272, 33121, 4114, 5307, 59122, 98140, Bt10, MIR162, MIR604, MON87411, MON87427, MON87460, MON88017, MON89034, TC6275, VCO-Ø1981-5
6.	Aerosol Beam Injection	HCEM485

*Source: ISAAA GM Crops Approval Database.*

Usually, the vectors used for transformation consist of a plant expression cassette along with other genetic elements in a bacterial plasmid. However, only the expression cassette is required for transgene expression and not the entire plasmid. In particle bombardment, T-DNA processing and integration steps are not involved. Therefore, the vector backbone is redundant and unnecessary. The so called vectors or plant transformation constructs are used in particle bombardment at best for operational handiness rather than experimental requirement. Fu et al. (2000) designed a strategy of particle bombardment using expression cassette only. In this strategy, all the vector sequences were removed prior to particle loading and the minimal cassette with gene of interest was removed from the plasmid. This linear cassette containing only transcription unit for gene of interest was used for transformation. The results demonstrated that transgene integration and expression were possible using just minimal cassettes also. As an added advantage, the resulting transgenic plants exhibited much simpler transgene integration patterns and lower copy numbers than the plants transformed with equivalent whole constructs. This approach was used for multiple gene transfer in plants by Agrawal et al. (2005), who performed particle bombardment with five separate marker gene cassettes. The majority of the transformed plants showed simple integration patterns with a high proportion of single-copy events, high transgene expression, and inter-generational transgene stability. Thus, the earlier belief that particle bombardment generates large, multi-copy events, prone to instability and silencing may not be true and the refinements in the particle bombardment technology, especially ‘clean DNA transformation’ (Agrawal et al., 2000, 2005) demonstrate the versatility and precision of this method (Altpeter et al., 2005). This development also opened possibility of development of marker free transgenic plants with multiple genes. Generation of high quality transgenic events, in terms of clean site of integration without disrupting any endogenous gene, getting single-copy insertions, and ensuring absence of any vector backbone, etc., is also important from regulation perspective. Refinements in transformation technologies that aid recovery of quality events are very much desirable.

### Silicon Carbide Whiskers

Silicon carbide whiskers are needle like structure having size of 20 μm in length. They penetrate cell wall and plasma membrane of target cell to transfer desired DNA and thus, the transformants are obtained (Southgate et al., 1998). In maize, non-regenerable variety Black Mexican Sweet was transformed by silicon carbide whiskers (Kaeppler et al., 1992). Fertile transgenic maize plants have been developed successfully from Type II callus and cell suspension culture using this method (Frame et al., 1994; Petolino et al., 2000). But this method has certain limitations, such as low transformation frequency and delivery of DNA only to fine cell aggregates. Like silicon carbide whisker, another physical method of gene delivery using an airgun apparatus has also been used for transient gene expression studies in maize. This apparatus utilizes compressed air from a commercial airgun to force macroprojectiles and DNA-coated tungsten particles.

### *Agrobacterium*-Mediated Transformation

*Agrobacterium tumefaciens* soil pathogen is a natural genetic engineer which has ability to transform plants. From last two decades, *A. tumefaciens* has been frequently used for transformation of dicot plants. In comparison to direct DNA transfer methods, *Agrobacterium*-mediated method has a major advantage that in this method low copy of relatively large DNA fragments can be integrated into host plant genome with minimum rearrangement. This results in high quality transgenic plants. Initially, it was supposed that this technique cannot be used for monocot plants. In mid 1980s, it was demonstrated that *Agrobacterium* mediated plant transformation can be employed for maize (Graves and Goldman, 1986; Grimsley et al., 1987). Gould et al. (1991) used shoots as target tissue for transformation. The major breakthrough came from *Agrobacterium*-mediated transformation of maize by Ishida et al. (1996), followed by transformation in other cereals with similar protocols. Ishida et al. (1996) used *Agrobacterium* strain having super-binary vector pTiBo542 containing *vir* genes to transform immature embryos. Since Ishida et al. (1996) gave basic *Agrobacterium*-mediated transformation protocol, it has been greatly improved. The improvements include heat pre-treatment, addition of copper and silver ions to co-cultivation medium and increase in co-cultivation period from 3 to 7 days. Effects of these changes were quite evident. Traditional *Agrobacterium*-mediated transformation can be limited by host specificity and inability of *Agrobacterium* to reach cells in target tissues. A new improved *Agrobacterium*-mediated transformation method has been developed that overcomes above mentioned barriers. It increases transfer of DNA in different plants. This technique is known as Sonication-Assisted *Agrobacterium*-mediated transformation (SAAT) which involves periodic exposure of target plant tissue to sonication waves in the presence of *Agrobacterium* (Trick and Finer, 1997).

### *In planta* Transformation

*In planta* transformation of *Arabidopsis* by vacuum infiltration of whole plants (Bechtold et al., 1993) and the floral dip (Clough and Bent, 1998) are now routinely used. However, similar protocols for maize are not feasible. In maize, Otha (1986) and Yang et al. (2009) have depicted the possibility of pollen-tube pathway mediated transformation. However, the transformation frequency was reported to be quite low and the screening of the transformants by PCR analysis was time-consuming. Abhishek et al. (2014b) developed a tissue culture independent protocol for *in planta* transformation in tropical maize by using plumular meristems of germinating seeds as explants and transforming them using *Agrobacterium* approach.

Most of the methods described above have been used for developing commercial transgenic events in maize. However, the maximum numbers of commercial transgenic events have been developed using particle bombardment, followed by *Agrobacterium*-mediated transformation (**Table 2**).

## Selection Systems

Selection system is very important for identification of transgenic events. It imparts a selective pressure which allows transformed cells to proliferate, while suppressing the growth or killing of the non-transformants. An effective selection system should have no negative impact on plant regeneration. Widely used selection systems in maize transformation are listed in **Table 3**.

**Table 3:** Different selection systems for generating maize transformants.

Category	Selectable marker gene	Selection agent(s)
Antibiotic resistance	Hygromycin phosphotransferase (*hpt*)	Hygromycin B
	Neomycin phosphotransferase (*npt II*)	Kanamycin, paromomycin, G418
Herbicide resistance	Aryloxyalkanoate dioxygenase (*aad-1*) from *Sphingobium herbicidivorans*	*R*-haloxyfop
	Maize acetolactate synthase/acetohydroxy acid synthase (*ALS/AHAS*)	Chlorsulfuron, imazethapyr
	Bialaphos resistance *(bar)*, phosphinothricin acetyltransferase (*pat)* from *Streptomyces hygroscopicus* and *Streptomyces viridochromogenes*	Phosphinothricin, glufosinate, bialaphos
	5-enolpyruvoylshikimate-3-phosphate synthase from *Agrobacterium* spp. CP4 (*epsps*)	Glyphosate
	5-enolpyruvoylshikimate-3-phosphate synthase from maize (*EPSPS*)	Glyphosate
	Protoporphyrinogen oxidase (*PPO*) from *Arabidopsis*	Butafenacil
Sugar metabolism	phosphomannose-isomerise (*pmi*); *man*A gene from *E. Coli*	Mannose

### Antibiotic Resistance

Neomycin phosphotransferase II gene *(nptII)* obtained from *Escherichia coli* Tn5 transposon and kanamycin were used in early maize transformation experiments. So far, 16 events of transgenic maize containing *nptII* have been commercialized. Another antibiotic selection system is based on hygromycin phosphotransferase *(hpt)* with hygromycin B as selection agent. Hygromycin inhibits single cell or small clusters of cells and their growth but large clumps of cells are less susceptible to antibiotic selection at later stages of transformation, which require high concentration of antibiotic that may have damaging effect to selected cells or plants. For this reason, *hpt* and hygromycin are now generally not used widely as selection system in maize and no *hpt* containing maize transgenic has been released ever.

### Herbicide Resistance

The use of herbicide resistance offers dual advantage of being a potent selectable marker as well as an important agronomic trait. The widely used selectable markers- *bar* and *pat* genes, isolated from *Streptomyces hygroscopicus* and *Streptomyces viridochromogenes*, respectively, both encode phosphinothricin acetyltransferase (PAT). PAT eliminates herbicidal activity of glufosinate (phosphinothricin) herbicides by acetylation. The *bar* gene was the first herbicide selectable marker gene which was used in selection of transformed maize cells. In maize, selections of callus using *bar* and *pat* selectable marker have been found to be more efficient than kanamycin. So far, in maize, 83 events have been commercialized with *pat* gene, five events with a synthetic (*syn*) version of *pat* gene and seven events with *bar* gene. Glyphosate resistance is an important trait for the control of broad spectrum weeds. The different forms of 5-enolpyruvoylshikimate-3-phosphate synthase *(epsps*) genes have been commonly used for glyphosate resistance. The *epsps* gene from three sources have been used in maize genetic engineering- the most widely used *CP4epsps (aroA:CP4*) gene obtained from *Agrobacterium tumefaciens* strain CP4; the mutated *mepsps* and the double mutant *2mepsps* gene obtained from maize itself and the *epsps grg23ace5* gene which was chemically synthesized based on the sequence of *epsps grg23* gene from soil bacterium *Arthrobacter globiformis*. Apart from *epsps*, glyphosate oxidoreductase *(gox)* gene- *goxv247* obtained from *Ochrobactrum anthropi* strain LBAA and glyphosate N-acetyltransferase *(gat)* gene*- gat4621* obtained from *Bacillus licheniformis* can be used to detoxify glyphosate. More than 90 events of transgenic maize containing glyphosate resistance have been released. Improved *gat* marker gave the transformation frequency of 64% (McCutchen et al., 2007). Mutants of acetolactate synthase (*als)*, also known as acetohydroxy acid synthase *(ahas*), confer resistance in transformants against ALS inhibitor family of herbicides such as sulfonylurea and imidazolinone (Le et al., 2010). In maize, mutants of *als/ahas* have been successfully used as selectable markers (Bernasconi et al., 1995). Protoporphyrinogen (*ppo*) gene is another robust selectable marker, coding for a double mutant which are resistant to butafenacil, which inhibits the activity of PPO enzyme which in turn results in protoporphyrin IX mediated light-dependent membrane damage (Li et al., 2003). Another gene, *aad-1* (also known as *Rdp A* gene) isolated from *Sphingobium herbicidivorans* encodes aryloxyalkonate dioxygenase which cleaves aryloxy phenoxypropionate (AOPP) herbicides specifically inhibiting the monomeric acetyl-CoA carboxylases from monocots. The *aad-1* gene has been efficiently used as selectable marker for maize transformants (Wright et al., 2010).

### Sugar Metabolism

Mannose-6-phosphate isomerase (PMI) encoding gene, *manA* allowed selection of maize transformants on mannose containing media (Negrotto et al., 2000; Wang et al., 2000). PMI have higher transformation frequency and powerful selection. Another sugar metabolism based positive selection system based on *xyl A* gene, similar to PMI, encoding xylose isomerase have been tested in maize and in this system, xylose is used as selection agent (Guo et al., 2007).

### Marker Free Transgenics

Huang et al. (2004) emphasized many variations in the approach for generating marker free transgenics. A strategy referred to as 2T-DNA transformation involves placing the selection marker and the gene(s) of interest on two separate T-DNAs. As the genes of interest and the selection markers are physically separate, their transfer and integration to the plant chromosome are mutually independent. In the cells that have received both the genes, the marker gene can be expected to integrate at an independent site from that of the gene of interest. The plants produced from such cells would produce segregant progenies in the next generation that may be free from the selectable marker but contain the gene of interest.

## Commercial Success of Maize Transgenics

So far, 143 different events of transgenic maize have been approved for commercial cultivation or food/feed use across 30 countries (with European Union counted as one country). The released events belong to six major trait groups- herbicide tolerance (121 events), insect resistance (115 events), modified product quality (12 events), pollination control system (6 events), and abiotic stress tolerance (4 events), with stacking of events being a common phenomenon. In 2015, out of 185 million ha of global maize area, 29%, i.e., 53.6 million ha was planted with maize cultivars with transgenic traits (James, 2015).

## Transformation for “New Breeding Techniques”

While the first transgenics in maize were commercialized about 20 years ago, there has been evolution of transgenic landscape in terms of techniques, regulation, and public perception. A set of new techniques, popularly termed “New Breeding Techniques” are rapidly evolving. Some of these, like, gene editing, cisgenesis, intragenesis, RNA-dependent DNA methylation, etc., would necessitate further fine tuning of the maize transformation work-flows.

### Cisgenics and Intragenics

In cisgenesis, the complete coding sequence (CDS) including introns of a gene originating from the sexually compatible gene pool of the recipient plant along with gene’s own promoter and terminator are used for transformation (Schouten et al., 2006). In this case, the cisgene should be used in its normal sense orientation only. In intragenesis, the full or partial CDS of genes originating from the sexually compatible gene pool of the recipient plant can be used in sense or antisense orientation. In this case, the promoter and terminator could originate from sexually compatible gene pool of the recipient plant and not necessarily from the ‘cisgene’ itself (Rommens et al., 2004). Since, both cisgenics and intragenics would be essentially “marker free,” transformation strategies directed at recovering marker-free plants, had to be essentially employed. In both these cases, particle bombardment using ‘clean DNA transformation’ may be employed. So far, there are no reports of cisgenic or intragenic maize under commercialization or advanced development.

### Gene Editing

Targeted genome modifications or gene editing with the help of site-specific nucleases (SSNs) have the potential to avoid many regulatory issues regarding transgenics. Site-specific nucleases include Zinc-finger nucleases (ZFNs), Transcription activator-like effector nucleases (TALENs), and Clustered regularly interspaced short palindromic repeats (CRISPR)/CRISPR-associated (Cas). ZFNs and TALENs are artificial proteins composed of a specific DNA-binding domain and DNA cleavage domain. In these approaches, double strand breaks (DSBs) are introduced at targeted sites in the DNA. The DSBs are immediately repaired by two mechanisms, *viz*. non-homologous end joining (NHEJ), which is a type of error prone repair and by Homology direct repair (HDR). With the help of these molecular repair processes, researchers have been able to disrupt specific genes either by inserting exogenous DNA elements into desired genomic sites or by introducing single-nucleotide substitutions. CRISPR together with Cas proteins form CRISPR-Cas system, is the newest genome modification technique. ZFN method has been used to modify endogenous loci in crop plants, like maize. The CRISPR/Cas9 system has been used for gene editing in crop plants like, rice, wheat, sorghum, tomato, tobacco, etc.; with few examples in case of maize as well (Feng et al., 2016). Transformation for gene editing may require co-bombardment of separate DNA vectors containing Cas9 and gRNA. However, stable integration and constitutive expression of gRNAs and Cas9 might lead to somatic mutations and generation of chimeric plants. In this scenario, gRNA may be delivered in form of *in vitro* synthesized RNA molecule, together with Cas9 as DNA construct. Bombardment of RNA molecules posses further challenges of optimization of particle preparation and gene gun operation protocols. Genetic transformation usually involves transgene integration into the host genome. However, introduction of genes without genomic integration is more desirable for HDR, and other transient expression requiring genome editing tools. Bombardment of single-stranded DNA has been used as one of the approach to evade template integration during HDR-mediated genome editing in maize (Svitashev et al., 2015). Refinements in *Agrobacterium*-mediated transformation and further development of RNA viruses and geminiviruses based transformation techniques may result in gene transfer protocols with superior genome editing properties (Altpeter et al., 2016). In targeted genome modification by SSNs, modifications performed in organisms’ endogenous gene to develop desired traits do not employ transgene of other species or organism. Targeted genome modified products are essentially mutation based products. Therefore, plants developed using these technologies may be treated at par with mutation breeding products and could be regulated as such. This would ensure faster commercialization and easier availability of this technology for the welfare of the farmers.

## Future Perspectives

This review attempted to provide a summary of the advances in maize transformation involving all available transformation methods. Despite significant progress made, the success rate of genetic transformation of maize is still insufficient. This is because of various limitations with the presently available maize tissue culture and transformation protocols. There is a need for further efforts to develop genotype-independent versatile maize transformation workflows that can be adapted in any laboratory. While there has been tremendous progress in fundamental research aimed at unraveling biological processes and pinning underlying genetic regulation in plants, the techniques of transformation have largely remained archaic. Improvement of transformation workflows that can lead to automation and increased throughput, represent biological as well engineering challenges. The particle bombardment technique could be further improved. The basic design and operation of the gene gun has remained almost same for the last 20 years. Advances in nanotechnology can be harnessed to develop new nano-based micro-projectiles which may cause minimal damage to target tissue and facilitate delivery in precise and clean manner. Greater focus is also required to study transformation responses of a wide range of tissues and genotypes. A plethora of genetic and epigenetic mechanisms have been found to modulate callus induction, differentiation, embryogenesis, and other developmental pathways, that are so crucial in a tissue culture cycle. However, little efforts have gone into employing endogenous predispositions in developmental biology through genetic manipulation or identification of natural or mutant genotypes with these predispositions for advancing tissue culture. Similarly, manipulation of infection-responsive host genes may lead to better maize transformation efficiency through *Agrobacterium*. Use of geminiviruses in maize transformation should also be explored as novel viral delivery systems. Maize transformation techniques have evolved from single cell approaches, like protoplast transformation and presently overwhelmingly rely on immature embryo tissues as explants. There might be a need to revisit protoplast transformation in light of greater automation potential that it can offer. The use of a plant transformation and genome editing robot was recently demonstrated in Bright Yellow 2 (BY-2) tobacco suspension cultures (Dlugosz et al., 2016). Similar approaches for maize transformation may be attempted. Only when maize transformation techniques become simpler, cheaper, and robust, the genetic modification technology would be used for greater public good in public institutions, especially in the developing world.

## Author Contributions

PA conceived the idea of review, provided inputs for specific sections, and edited the final draft. The primary manuscript was written by PY, AA, and RS. IS, TK, and AP provided specific comments and improved the draft. All the authors read and approved it for publication.

## Conflict of Interest Statement

The authors declare that the research was conducted in the absence of any commercial or financial relationships that could be construed as a potential conflict of interest.
